# Studies on the Interaction of Poly(phenylene methylene) with Silver(I) and Hexacarbonylchromium(0)

**DOI:** 10.3390/polym14173465

**Published:** 2022-08-25

**Authors:** Xavier H. Guichard, Andreas Braendle, Markus Niederberger, Walter Caseri

**Affiliations:** Department of Materials, ETH Zürich, 8093 Zürich, Switzerland

**Keywords:** poly(phenylene methylene), coordination, silver(I), tricarbonylchromium(0), photoluminescence, homoconjugation

## Abstract

Complexes of poly(phenylene methylene) (PPM) with silver(I) ions and tricarbonylchromium(0) moieties, respectively, were synthesized. ^13^C NMR spectra indicate interaction of phenylene groups with silver(I) and chromium(0), and peak broadening implies dynamic behavior of the silver(I) complexes, with all phenylene groups temporarily involved in coordination, in contrast to the chromium complexes. About 5–10% of the phenylene groups are coordinated to metal atoms. ^1^H NMR and IR spectra, in the case of chromium(0), and the solubility of silver salts in the presence of PPM provide further evidence of coordination. The complexes are soluble in chloroform, but the silver complexes decay in tetrahydrofuran (second-order kinetics were observed in an example). The photoluminescence (fluorescence) of PPM is maintained upon complexation, although coordination of silver(I) seems to favor the so-called blue phase of PPM relative to the green phase by a factor of approximately two in PL spectra. The pronounced absorption of the tricarbonylchromium(0) units interferes with the blue phase, which almost disappears at a concentration of 50 mg/mL in PLE spectra, whereas the emission maximum of the green phase is hardly affected. This leads to a confinement of the emitted wavelength range of PPM. Thus, the perceived optical emission of PPM can be modified by coordinated entities.

## 1. Introduction

Poly(phenylene methylene) (PPM) is an aromatic hydrocarbon polymer with the formula (C_6_H_4_-CH_2_)_n_ that has been the subject of research for decades [1,2,3,4,5,6]. It is thermally very stable (thermal decomposition above 400 °C) [4,5,7,8,9], has recently attracted attention with respect to corrosion protection [10] and shows photoluminescence (fluorescence) [4,6,11,12,13]. The fact that photoluminescence occurs in spite of the interrupted conjugation of the aromatic units was recently studied in detail [6,13]. As a result, the origin of the photoluminescence was attributed to homoconjugation [6,13], which is defined by IUPAC as the overlap of orbitals that are part of two π-systems and which are separated by a non-conjugating unit (for instance, a methylene group) [14]. Although this phenomenon is well-established, it is relatively rare, in particular in polymers.

Aromatic units (such as those in PPM) can also coordinate to metal centers, and soluble complexes of PPM and tricarbonylchromium(0) have been reported, although with low molar mass (number average molar mass (M_n_) below 3000 g/mol) [15]. However, the photoluminescence of the resulting material was not addressed. Accordingly, we examined this aspect in the present study but with PPM of substantially higher molar mass (M_n_ = 16,000 g/mol), as the peak maxima in photoluminescence spectra of PPM depend on its molar mass up to an M_n_ of about 10,000 g/mol and then remain essentially constant at higher molar masses [6].

In addition, we investigated whether phenylene units in PPM also coordinate to silver(I) ions, as silver(I) can basically coordinate to arenes [16,17], and we examined the related photoluminescence. Notably, arenes interact with silver(I), commonly by η^2^ coordination, whereas η^6^ coordination usually occurs with tricarbonylchromium(0) [16].

Due to the peculiarity of the photoluminescence of PPM, some explanations are required in order to follow the results presented below. A characteristic attribute of PPM is the appearance of two photoluminescent species [6]. Although there is a certain overlap of their emission wavelengths (400–480 nm and 450–560 nm) the former emits predominantly in the blue and the latter in the green region. Accordingly, they were designated as blue phase and green phase, respectively. The origin of the difference between these two phases is hitherto not clear. It has been hypothesized that the phases might correspond to different conformations or different substitution patterns of the phenylene units [6], as PPM generally contains ortho-, meta- and para-substituted phenylene units [1,2,3,11,18,19,20,21,22].

**Figure 1:**
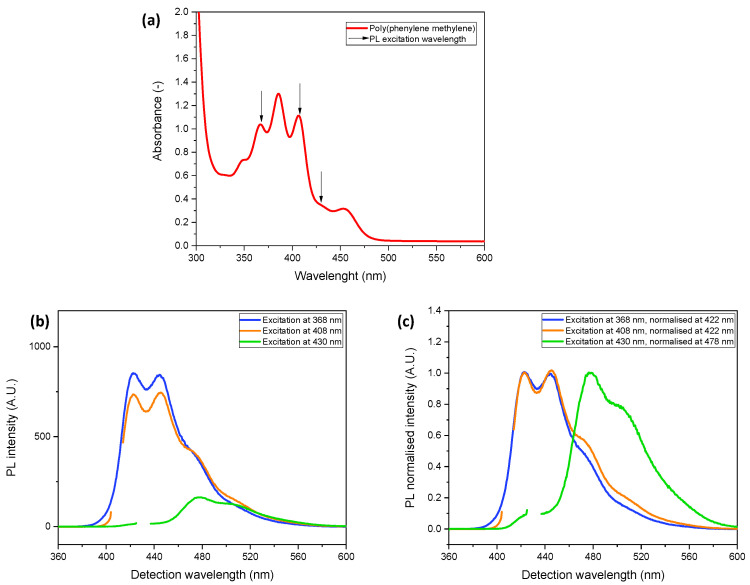
(**a**) UV-vis spectrum of PPM (30 mg/mL in chloroform); (**b**) PL spectra of PPM excited at 368 nm (blue curve), 408 nm (orange curve) and 430 nm (green curve); (**c**) normalized PL spectra related to (**b**).

**Figure 2:**
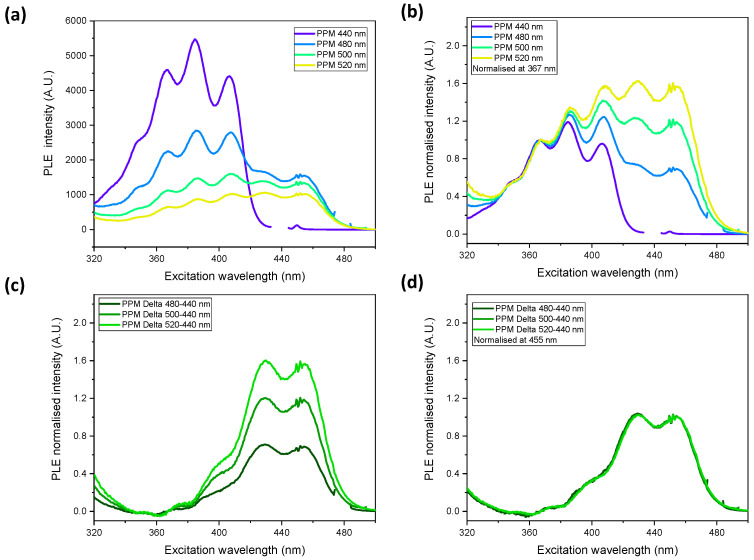
(**a**) PLE spectrum of PPM at detection wavelengths of 440 nm (violet curve), 480 nm (blue curve), 500 nm (green curve) and 520 nm (yellow-green curve); (**b**) normalized PLE spectra related to (**a**); (**c**) PLE spectra of (**b**) after subtraction of the detection wavelength at 440 nm (blue phase); (**d**) normalized PLE spectra related to (**c**).

The two phases are evident in UV-vis, photoluminescence (PL) and photoluminescence excitation (PLE) spectra, as illustrated in Figure 1 and Figure 2 with the example of the PPM batch used in this study. The UV-vis absorption spectrum (Figure 1a) shows several maxima at higher energy (about 350 nm, 367 nm, 386 nm and 407 nm), which are attributed to the blue phase and an absorption maximum, with a shoulder at lower energy, which is characteristic of the green phase (453 nm, shoulder around 530 nm) [6].

The PL spectrum obtained upon excitation at 368 nm represents essentially the blue phase, which still dominates with excitation at 408 nm, whereas excitation at 430 nm results virtually in the spectrum as that of the green phase [6]. This is shown in Figure 1b, which also reveals that the intensity of the green phase is lower than that of the blue phase. For improved comparison of the spectral shapes, the spectra are also displayed with the peak maximum normalized to 1 at specific wavelengths, as indicated in Figure 1c. Thus, it becomes evident that upon excitation at 408 nm, the PL spectrum is already slightly influenced by the green phase. Peak maxima emerge at 423 nm and 445 nm for the blue phase and at 477 nm with a shoulder around 500 nm for the green phase.

The PLE spectrum detected at 440 nm provides essentially the spectrum of the blue phase, whereas detection at higher wavelengths up to 520 nm leads to a superposition of the spectra of the blue and green phases [6]. Figure 2a implies that the PLE intensity of the green phase is lower than that of the blue phase. The differences in the spectral shapes at different detection wavelengths, associated with the relative evolution of the green phase at higher detection wavelengths, is obvious from Figure 2b, which displays spectra normalized to the first PLE maximum emission wavelength of the blue phase at 367 nm. When the normalized blue phase spectrum (excitation at 440 nm) is subtracted from the PLE spectrum at detection wavelengths of 480 nm, 500 nm and 520 nm, spectra of the same shape are obtained (Figure 2c). The shapes of these spectra are virtually identical, which becomes obvious upon normalization to the peak at 455 nm (Figure 2d). Hence, the spectra in Figure 2c,d are assumed to represent the spectrum of the green phase.

## 2. Materials and Methods

### 2.1. Chemicals

Chemicals were purchased from the following companies: Dichloromethane (99 + %), chloroform (99.8%), pentane (99.0%), decalin (98%, cis and trans), methanol (99.8%), benzyl chloride (99%) and SnCl_4_ (99%) from Sigma-Aldrich (Buchs, St.Gallen, Switzerland); AgSO_3_CF_3_ (99%) from Fluorochem (Hadfield, Glossop, UK); AgClO_4_ (97%) and [Cr(CO)_6_] (99%) from ABCR (Kasru, Baden-Wuerttemberg, Germany); and dry THF (99.5%) over a molecular sieve from Fisher Scientific (Schwerte, Nordrhein-Westfalen, Germany). All chemicals were used as received. 

### 2.2. Synthesis of PPM

PPM was synthesized by polymerization of benzyl chloride with SnCl_4_ as a catalyst, similar to a procedure described previously [22]. A four-necked 350 mL flask was evacuated and flushed several times with nitrogen before adding the reactants. A constant flow of nitrogen (1.045 L/h) was applied to avoid oxidation and to flush hydrogen chloride out, which evolved during the reaction. The flow was controlled with a Q-flow flow controller from Vögtlin (Muttenz, Canton of Basel-Landschaft, Switzerland). The gases were passed through a neutralizing solution of NaOH in water. The neutralizing solution consisted of 500 mL water, to which a total amount of 49.289 g of solid NaOH was slowly added in small portions to keep the pH value at about 12. The volume of the employed benzyl chloride amounted to 140 mL (1.216 mol) and that of SnCl_4_ to 0.9 mL (12.2 mmol). The resulting mixture was heated to 80 °C under stirring, and the start of the reaction was indicated by HCl evolution and a red color of the reaction mixture. One hour later, the temperature was increased to 120 °C, and two hours later, to 160 °C to lower the viscosity and to improve the stirring. The reaction was then continued overnight. Afterwards, heating was stopped, and the reaction mixture was cooled down to room temperature. When the mass had solidified, 270 mL of tetrahydrofuran was added under stirring to dissolve the polymer. The solution was then added dropwise to 3.9 L of methanol under vigorous stirring to precipitate the polymer. Stirring was continued for several hours to obtain a fine polymer powder. Finally, the powder was filtered and dried under vacuum (3 × 10^−3^ bar at 50 °C) for 72 h. The yield amounted to 102.7 g (94%). The ^1^H and ^13^C NMR spectra corresponded to those reported earlier [22], the number average molar mass (M_n_) was 16,000 g/mol and the weight average molar mass (*M*_w_) was 41,000 g/mol, as determined by gel permeation chromatography as described previously [22]. This batch of PPM was used for all experiments.

### 2.3. Preparation of PPM-Ag^+^ Complexes

In the following experiments, the light in the laboratory was reduced to a minimum, as silver complexes, in general, are subject to a certain sensitivity to light. Under a slight nitrogen stream, 400.0 mg (1.56 mmol) AgSO_3_CF_3_ or 322.8 mg (1.56 mmol) AgClO_4_ was suspended in 100 mL of chloroform or dichloromethane, resulting in a colorless dispersion. Separately, 1.600 g PPM was dissolved in 50 mL of the respective solvent (chloroform or dichloromethane). The yellow PPM solution was added dropwise by a dropping funnel to the suspension of silver salt within 2 h, whereupon the silver salt dissolved, and a pink or reddish color developed (when the PPM solution was added too fast, a pink layer formed on the surface of the silver salt grains, and the silver salt did not dissolve any further). When the silver salt was completely dissolved, the solvent was removed by a rotary evaporator, and the remaining pink or reddish solids were dried under reduced pressure (3 mbar) at 50 °C for 14 h. Because no loss of the starting materials was possible during this procedure, the yield was essentially quantitative. The products were stored under protection from light. Analytical data are provided and discussed below in the Section 3.

### 2.4. Preparation of PPM-Cr^0^ Complexes

The preparation of tricarbonylchromium(0) complexes with low-molar-mass PPM was described three decades ago [15]. However, in this procedure, green chromium complexes formed as reaction side products, and we found that traces of such compounds gave rise to problems during NMR spectroscopy due to their obvious paramagnetic nature. Additionally, the filtration process through silica gel described in this procedure is problematic with PPM of high molar mass, such as that used in our study, due to adsorption. Therefore, we developed a new procedure without formation of green (paramagnetic) chromium species and that does not require filtration trough silica gel.

**Figure 3:**
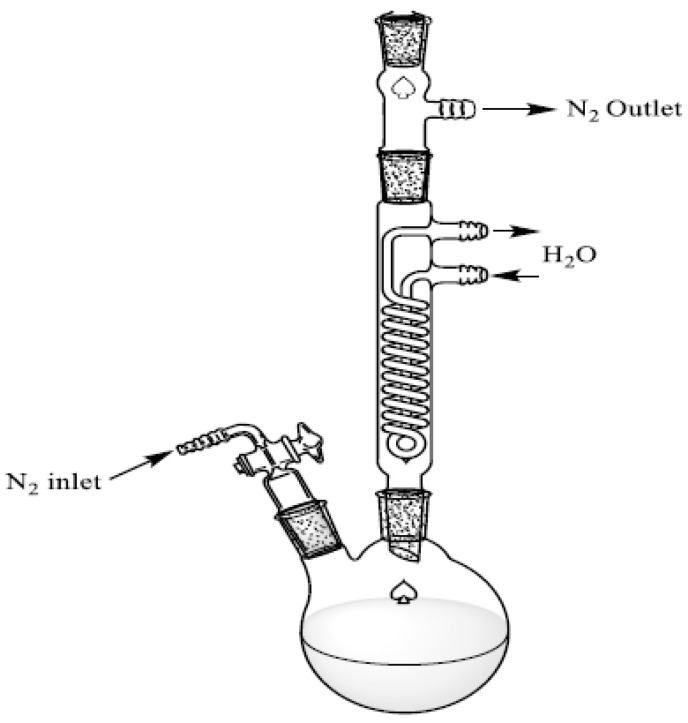
Experimental setup for the synthesis of PPM with coordinated tricarbonylchromium(0) entities.

The glassware was dried at 120 °C under vacuum (20 mbar) overnight, and the reaction vessel was filled with nitrogen. Subsequently, care was taken to ensure that a valve was open to release the carbon monoxide evolving upon conversion of [Cr(CO)_6_]. It was necessary to rinse the setup with a controlled flow of nitrogen throughout the reaction (0.45 L/h). Next, 502.3 mg of hexacarbonylchromium(0) and 1009.4 mg of PPM were added to a 50 mL two-neck flask. Then, the setup (Figure 3) was evacuated and flushed with nitrogen, and this procedure was repeated twice. Thereafter, 20 mL of decalin and 5 mL of THF were added through the upper septum. The solvents were used as purchased (dry and air-free sealed septum bottles). The temperature was raised to 120 °C, and the reaction mixture started to reflux. An increase in temperature within the reaction flask was occurring throughout the reaction due to the loss of THF. In order to balance this effect, small amounts of THF were added periodically through the upper septum (approximately 2 mL every 8 h). After 24 h, the reaction flask was cooled to room temperature, one connection was unplugged and the viscous liquid was extracted with a syringe to separate it from minor amounts of solids at the bottom of the flask. The reaction mixture was then precipitated into 70 mL of pentane and centrifuged, the liquid portion was removed with a syringe and the wet solid was redissolved in 2 mL THF. This operation was repeated three times by precipitating the THF solution in 35 mL of pentane to remove all the decalin from the product. Finally, the THF and pentane were removed from the remaining concentrated mixture by evaporation at reduced pressure and moderate temperature (30 mbar and 60 °C for 14 h). Thereafter, 960.0 mg of a deep yellow product was obtained. The chromium/polymer compound was stored in a dark environment. The analysis of the complex is reported below in the Section 3.

### 2.5. Spectroscopic Analyses

Infrared (IR) spectra were measured with the attenuated total reflection (ATR) technique using a diamond crystal on a Bruker Alpha FT-IR spectrometer (Billerica, MA, USA). ^1^H and ^13^C NMR spectra were recorded with a Bruker AV300 MHz instrument (Billerica, MA, USA), applying 128 scans for ^1^H NMR and 4096 scans for ^13^C NMR spectra. Deuterated chloroform and deuterated tetrahydrofuran were employed as solvents. As [Cr(CO)_6_] in the dissolved state is readily oxidized by air and because NMR spectra are sensitive to even slight amounts of oxidized chromium compounds, the NMR samples were prepared under an inert atmosphere, sealed with parafilm and immediately inserted into the spectrometer to yield unperturbed NRM signals. UV-visible absorption spectra of polymer solutions were recorded on a Jasco V-660 device (Pfungstadt, Darmstadt, Germany). Transmission spectra were measured in quartz cuvettes of 1 cm path length. Photoluminescence emission (PL) and excitation (PLE) spectra were measured using a Jasco FP-8500 device (Pfungstadt, Darmstadt, Germany). Solutions were placed in 10 mm × 1 mm quartz cuvettes, with the thin edge of the cuvette positioned at an angle of 45° to the incoming excitation beam. The short path length of the cuvettes reduced effects of self-absorption.

## 3. Results and Discussion

### 3.1. Complexes of PPM with Silver(I) Salts

**Figure 4:**
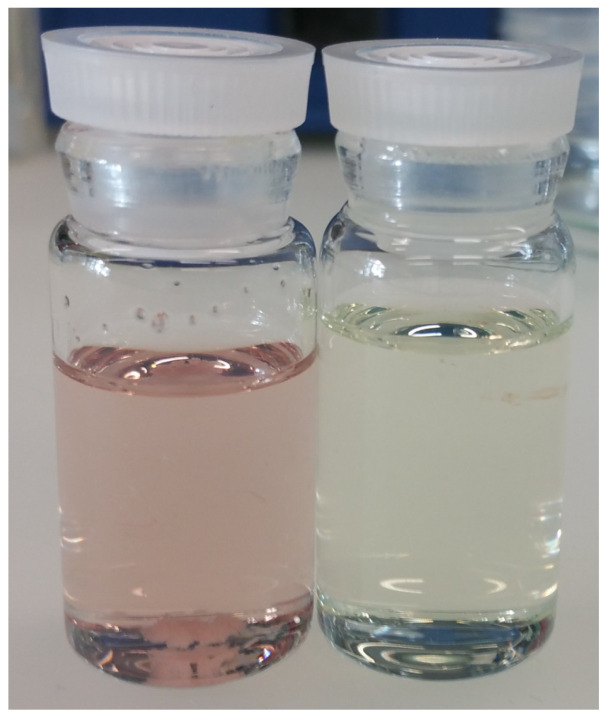
Typical color of a PPM-silver(I) solution (left vial) compared to the color of a PPM solution (right vial) in chloroform.

Upon slow addition of a PPM solution (yellow) to a suspension of AgSO_3_CF_3_ or AgClO_4_ in chloroform or dichloromethane under a nitrogen atmosphere, the respective silver salts dissolved, and a pink or reddish color developed (Figure 4). A ratio of Ag^+^/(phenylene group of PPM) of 1:10 was found to be preferrable, as the dissolution process is incomplete at higher ratios. As silver salts themselves are insoluble in those solvents, the dissolution of the silver salts in presence of PPM indicates the formation of silver complexes with the polymer, which is supported by the color change. Pink or reddish products were isolated upon evaporation of the solvents; related IR spectra are shown in Figure S1 of the Supporting Material, whereas Section 2 of the Supporting Material with Figures S2 and S3 reveals that IR spectroscopy can be used for quantitative determination of silver salts in compositions with PPM, with the example of PPM-AgCF_3_SO_3_. A reddish color was also preserved upon dissolution of those products in chloroform (e.g., stirring for 6 h under ambient conditions with a concentration of 50 mg/mL), whereas solutions in tetrahydrofuran were essentially colorless. In contrast to chloroform, tetrahydrofuran is a suitable solvent for both AgSO_3_CF_3_ and AgClO_4_. Accordingly, the respective colors in tetrahydrofuran and chloroform may indicate that silver-PPM complexes are stable in chloroform but decay in tetrahydrofuran, as shown below.

#### 3.1.1. Evidence of Formation of Coordination Compounds

**Figure 5:**
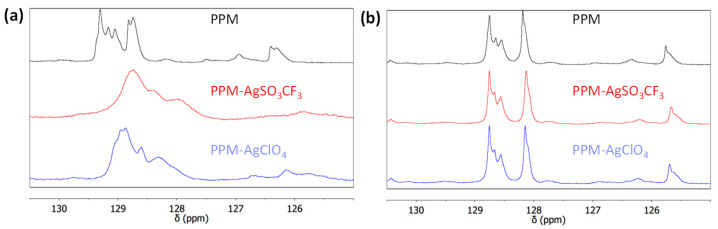
Section of ^13^C NMR spectra of the aromatic region of PPM alone and PPM with AgSO_3_CF_3_ or AgClO_4_ in (**a**) chloroform and (**b**) THF.

In line with the colors described above, coordination of silver to phenylene groups of PPM in chloroform but not in tetrahydrofuran is indicated by ^13^C NMR spectra (Figure 5 and Figure S4 in the Supplementary Material). Whereas ^13^C NMR spectra of PPM in the presence of either of the two silver salts did not change significantly in tetrahydrofuran, in chloroform, the signals of the phenylene groups of PPM (125 ppm–130 ppm) became broader and shifted to higher fields, both with AgSO_3_CF_3_ and AgClO_4_. The observed shifts of approximately 0.5 ppm–1 ppm are in line with silver(I)-arene complexes, although shifts to higher fields [23] might be less common than shifts to lower fields [23,24]. The spectra differ to some extent for the two counterions; peak maxima were found at 128.7 ppm, 128.4 ppm (shoulder) and 128.0 ppm (shoulder) with AgSO_3_CF_3_ and at 129.0 ppm, 128.9 ppm, 128.6 ppm and 128.3 ppm with AgClO_4_. These differences are possibly due to the interaction of the coordinated silver(I) ions with their counterions. Peak broadening indicates a dynamic equilibrium, which is in agreement with the fact that aromatic peaks of PPM disappeared entirely, although the amount of silver ions was not sufficient for coordination of all phenylene units (molar ratio of Ag^+^:phenylene units = 1:10) [25]. Thus, all phenylene groups are involved in coordination, although not at the same time as the silver(I) ions are rapidly exchanged. Dynamic processes have been reported for silver(I)-arene complexes [16,24], which readily arise due to η^2^ coordination of Ag^+^ to arenes [16].

As a side note, in ^1^H NMR spectra (Figure S5), the aromatic peaks of PPM are very broad and not resolved, as previously reported [22]; accordingly, changes in the spectra in the presence of silver salt were not evident.

#### 3.1.2. Optical Properties

**Figure 6:**
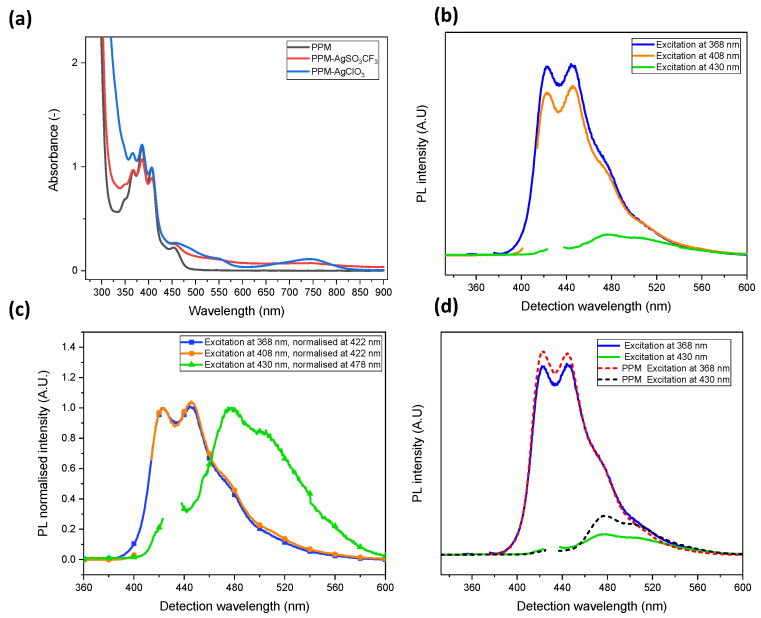
(**a**) UV-vis spectra of PPM alone and PPM with silver(I) salts (0.1 Ag^+^ per phenylene group, 30 mg/mL in chloroform); (**b**) PL spectra of PPM-AgCF_3_SO_3_ in chloroform excited at 368 nm, 408 nm and 430 nm; (**c**) normalized spectra related to (**b**); (**d**) comparison of PL spectra of PPM alone and PPM-AgCF_3_SO_3_ (intensities adjusted to that of the respective blue phase).

Compared to PPM alone, UV-vis-NIR absorption spectra of PPM-AgCF_3_SO_3_ in chloroform reveal a weak but relatively steady increase in absorbance in the visible wavelength range, whereas a weak and very broad absorption maximum at 739 nm and a shoulder at 540 nm emerge in the product with AgClO_4_ (Figure 6a), i.e., as in the ^13^C NMR spectra, a certain influence of the counterion emerges in UV-vis spectra.

The polymers with coordinated silver(I) were still fluorescent. PL spectra of PPM-AgCF_3_SO_3_ upon excitation at 368 nm and 430 nm (Figure 6b) represent the blue and green phases, respectively, of PPM alone (see Introduction). However, upon excitation at 368 nm and 408 nm, the PL spectra of the PPM-silver(I) samples show only minor differences above 460 nm (particularly evident in the normalized spectra in Figure 6c), in contrast to PPM alone, for which slightly but distinctly higher intensities arise upon excitation at 408 nm (Figure 2d) due to superposition of the green phase (see Introduction). Hence, it appears that the green phase is discriminated relative to the blue phase by the presence of silver(I) ions. This is also evident upon excitation at 368 nm (blue phase) and 430 nm (green phase), when the PL spectra of PPM with and without silver(I) are scaled by one constant (by adjusting to the same value at 368 nm with and without silver) (Figure 6d). In the presence of silver(I), the intensity of the absorption maximum of the green phase was about a factor of two lower relative to that of the blue phase.

**Figure 7:**
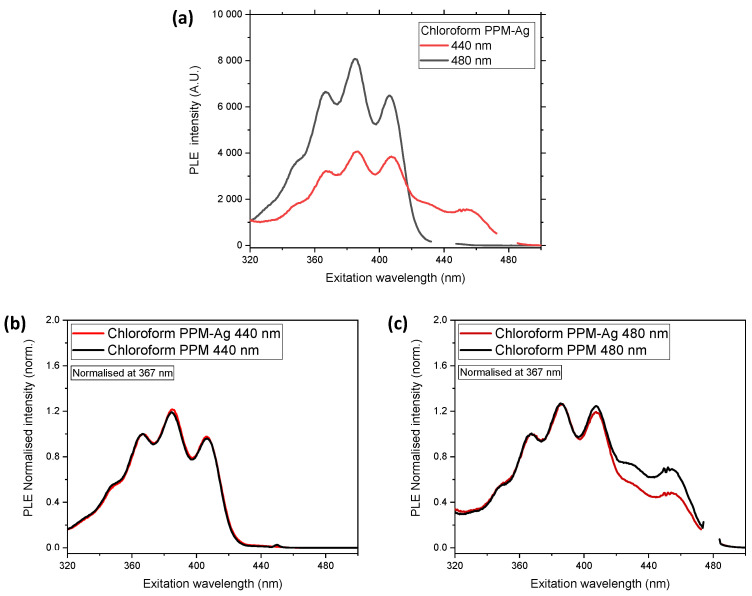
PLE spectra of PPM and PPM-AgCF_3_SO_3_ (0.1 Ag^+^ per phenylene group, 50 mg/mL in chloroform), (**a**) PPM-AgCF_3_SO_3_ detected at 440 nm (black curve) and 480 nm (red curve); (**b**) and (**c**) normalized spectra related to (**a**) and those of PPM at 440 nm and 480 nm, respectively.

A relative decrease in the green phase was also observed in PLE spectra (Figure 7). At a detection wavelength of 440 nm, the PLE spectrum represents the blue phase (see Introduction), whereas the normalized spectra of the Ag-PPM complex and PPM alone are virtually identical (Figure 7b). However, at a detection wavelength of 480 nm, which leads to a PLE spectrum consisting of a superposition of the blue and green phases, the intensity of the PLE spectrum of the Ag-PPM complex is lower than that of PPM alone at excitation wavelengths above ca. 420 nm (Figure 7c). As the influence of the green phase becomes significant at higher wavelengths (see Introduction), the decrease in intensity implies a reduction in the green phase relative to the blue phase under the influence of silver(I) ions. It cannot be ruled out that this preference of the blue phase might be associated with the dynamic behavior of the related complexes; however, because the difference in the nature of the blue and the green phases is not known, a definite declaration cannot be provided.

#### 3.1.3. Decay of Silver(I) Complexes in THF

**Figure 8:**
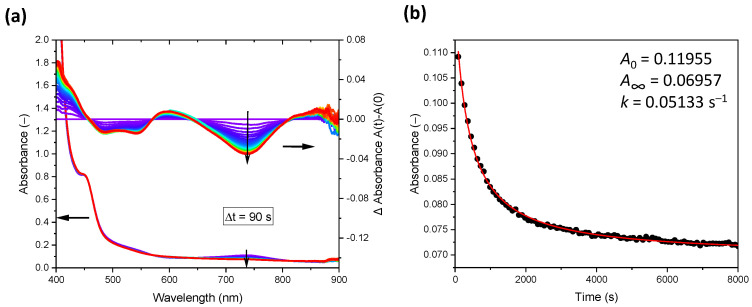
(**a**) Evolution of UV-vis spectra of the PPM-AgClO_4_ in THF, including difference spectra (right ordinate), with time intervals of 90 s between the individual measurements (0.1 Ag^+^ per phenylene group, 90 mg/mL); (**b**) Kinetic analysis of the evolution of the absorbance at 739 nm related to the spectra displayed in (**a**) fit to second-order kinetics (see text) between 0 s and 8000 s (red curve).

Although the absorbance at the absorption maximum at 739 nm in the PPM-AgClO_4_ solutions is only small, because the PPM-Ag^+^ complex had already decayed during preparation of the solution until insertion into the spectrometer, this band could still be used to follow the decay of the related silver(I)-phenylene species in THF (Figure 8).

The effective absorbance of the silver-phenylene species is the difference of the absorbance (*A*) and the background absorbance at infinite time (*A*_∞_). The temporal course of this difference (i.e., *A* − *A*_∞_) is proportional to the temporal course of the concentration of the silver(I)-phenylene species according to the Bouguer–Lambert–Beer law. Accordingly, the kinetic order of the decay is reflected directly by the temporal course of *A* − *A*_∞_. This course follows that of common second-order kinetics, i.e.,

(1) $$A-A_{\infty }=\frac{A_{0}-A_{\infty }}{1+k\left(A_{0}-A_{\infty }\right)t}$$

where *t* is the time, *A*_0_ is the absorbance at *t* = 0 s and k is the rate constant related to *A* − *A*_∞_ (Figure 2b, which also contains the fitted values of *A*_0_, *A*_∞_ and k). As the value of *A*_∞_ at 739 nm essentially corresponds to that of the baseline, the difference between *A* and *A*_∞_ is proportional to the concentration of the complex with coordinated silver(I). Therefore, the concentration of this complex is a function of *t*^−1^, thus reflecting the observed second-order kinetics. Hence, the rate-determining step in the decay of the silver species involves two species, e.g., two silver(I)-complex units or one silver(I) complex unit and one counterion if the concentration of counterions accessible to the silver(I) ions in the complexes is equal to the concentration of the complexed silver(I) ions (possibly due to the balance of electrostatic attraction between the oppositely charged ions). The concentration of THF, as the solvent, is virtually constant and therefore does not contribute to second-order kinetics. A mechanism of the decay of the silver(I)-complex with a simple break of the silver(I)-phenylene bond as the rate-determining step would be expected to lead to first-order kinetics. However, the above considerations are only tentative, as we do not have enough information to propose a detailed mechanism to explain the second-order kinetics of the decay of the silver(I) complexes. Second-order reactions of silver-polymer complexes were previously reported in a different context [26].

### 3.2. Complexes of PPM with Tricarbonylchromium(0)

Complexes of PPM and tricarbonylchromium(0) units were synthesized by reaction of PPM with [Cr(CO)_6_] in a 1:4 mixture of decalin and tetrahydrofuran (THF) at 120 °C for 24 h in an inert gas atmosphere, thus avoiding the formation of green chromium side products as reported elsewhere [15]. The yellow product thusly obtained appeared to be sensitive to sunlight (and in solution also to air). Accordingly, the product was stored in the dark.

#### 3.2.1. Evidence for Formation of Coordination Compounds

**Figure 9:**
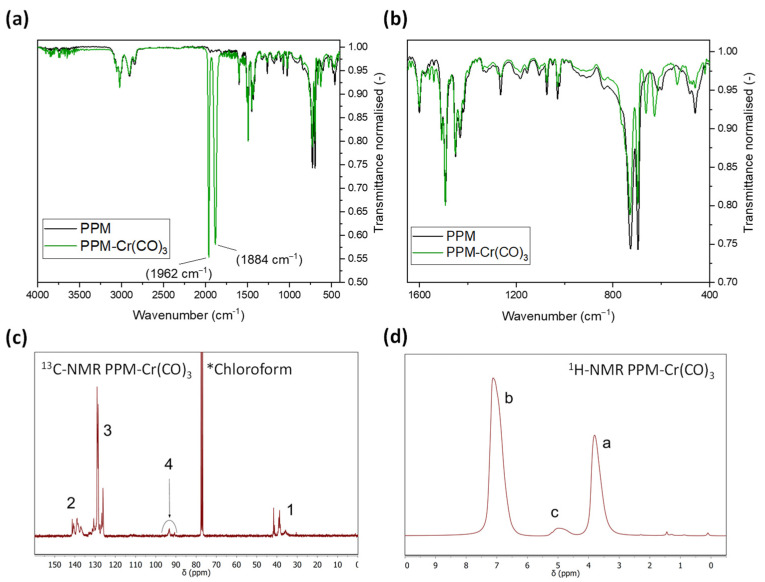
(**a**,**b**) IR spectra of PPM (black curve) and PPM-Cr(CO)_3_ (green curve, normalized at 1493 cm^−1^) for two different spectral ranges; (**c**) ^13^C NMR spectrum of PPM-Cr(CO)_3_ in d^1^-chloroform (200 mg/mL, signal groups 1, 2 and 3 essentially coincident with PPM alone, signal group 4 new); (**d**) ^1^H NMR spectrum related to (**c**) (signals a and b essentially coincident with those of PPM alone, signal c new).

Infrared (IR) spectra of the yellow product show the common signals of PPM, in addition two strong signals at 1962 cm^−1^ and 1884 cm^−1^ (Figure 9 and Figure S1). These signals are in the range of those of C≡O stretching vibrations of related complexes with low-molar-mass PPM [15] and with those of [Cr(η^6^-1,x-dimethylbenzene)(CO)_3_] with x = 1, 2 or 3 [27], which might serve as low-molar-mass model compounds comprising methyl instead of methylene groups as in PPM. Notably, the frequencies of the [Cr(η^6^-1,x-dimethylbenzene)(CO)_3_] complexes did not differ significantly from each other, i.e., it is not expected that tricarbonylchromium(0) complexes with ortho-, meta- and para-substituted phenylene units can be distinguished in the IR spectra of the PPM complexes. Two other smaller peaks at 663 cm^−1^ and 630 cm^−1^ arise in the PPM-chromium(0) complex, which are in the range of MCO π-type vibrations of [Cr(η^6^-arene)(CO)_3_] complexes [28]. Because no unambiguous shifts of bands of PPM alone could be detected (as in the cases with silver salts, spectra not shown), it is likely that only a minority of the aromatic rings of PPM is involved in the coordination to chromium, in line with the NMR spectra reported below. As a side note, the starting material [Cr(CO)_6_] only shows one band at 1988 cm^−1^ [29], i.e., there is no evidence of the presence of this compound after exposure to PPM.

In ^1^H and ^13^C NMR spectra, small but distinct peaks emerge at approximately 5 ppm (broad) and 93 ppm, respectively (Figure 9). The chemical shifts of these new peaks correspond to those of aromatic H and C atoms, respectively, of phenylene groups in low-molar-mass PPM coordinated to tricarbonylchromium(0) [15], in agreement with other aromatic units coordinated to tricarbonylchromium(0) [27,30,31,32]. Evaluation of the integrated intensities of the free and complexed phenylene units in ^1^H NMR spectra lead to the conclusion that 5.5% of the phenylene groups are coordinated to tricarbonylchromium(0) moieties. Because the signals of the non-coordinated carbon atoms of the phenylene groups are clearly visible and, moreover, considerable peak broadening was not observed, in contrast to the silver(I) complexes described above, it appears that the chromium complexes are not dynamic.

#### 3.2.2. Optical Properties

**Figure 10:**
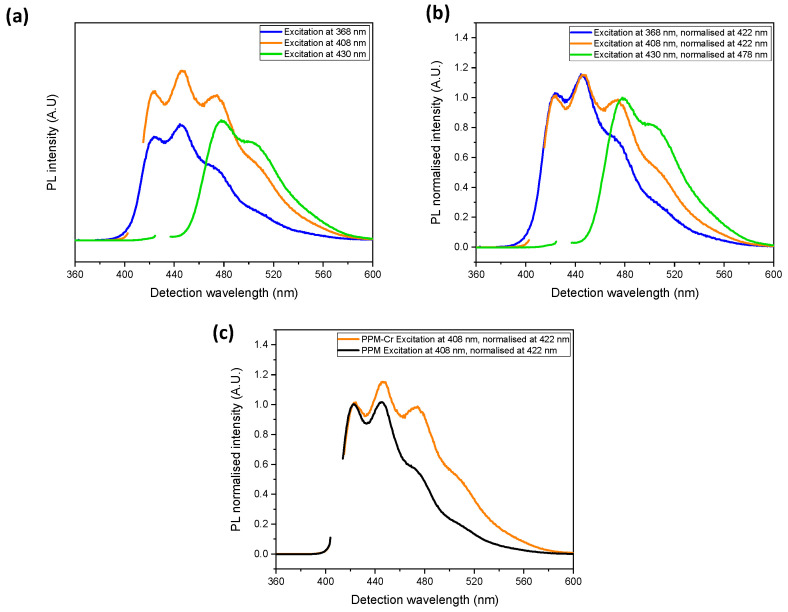
PL spectra (50 mg/mL) in chloroform, (**a**) PPM-Cr(CO)_3_ (0.055 Cr per phenylene group), excitation at 368 nm (blue curve), 408 nm (orange curve) and 430 nm (green curve); (**b**) normalized spectra related to (**a**); (**c**) normalized spectra with excitation at 408 nm of PPM (black curve) and PPM-Cr(CO)_3_ (orange curve).

The PPM-Cr(CO)_3_ product was fluorescent (Figure 10). However, a strong absorption of light in the UV region has to be considered, which is characteristic of coordinated tricarbonyl chromium-arene complexes [33] (at the respective absorption maxima, extinction coefficients of Cr(CO)_3_-arene complexes [33] are three orders of magnitude higher than that of PPM [6]). Thus, the absorption peaks of PPM alone were virtually covered in UV-vis spectra. Moreover, the absorption of the tricarbonyl chromium-arene units can interfere with the photoemission of PPM, in particular at higher energies, i.e., with regard to the blue phase. Upon comparison of PL spectra of PPM-Cr(CO)_3_ and PPM alone (Figure 10a,b vs. Figure 1b,c, respectively, and Figure 10c) the intensity of the green phase (higher-emission wavelengths) relative to the blue phase is distinctly higher in the PPM-Cr(CO)_3_ complex than in PPM alone, as evidenced in general by the higher relative intensities at higher wavelengths (i.e. lower energy) in the spectra of the PPM-Cr(CO)_3_ complex (Figure 10a,b vs. Figure 1b,c, respectively, and Figure 10c).

**Figure 11:**
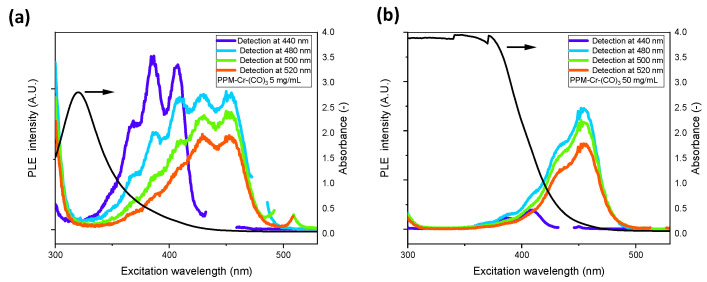
PLE spectra of PPM-Cr(CO)_3_ (0.055 Cr per phenylene group) in chloroform for emissions detected at 440 nm (dark blue curve), 480 nm (light blue curve), 500 nm (green curve) and 520 nm (red curve): (**a**) 5 mg/mL concentration; (**b**) 50 mg/mL concentration.

A pronounced discrimination of the blue phase relative to the green phase in PPM-Cr(CO)_3_ complexes compared to PPM alone is also evident in PLE spectra of PPM-Cr(CO)_3_ (Figure 11 vs. Figure 2). The influence of the absorbance caused by the tricarbonylchromium(0) unit is clearly evident in Figure 11, which shows PLE spectra, together with UV-vis absorption spectra, for two different concentrations. At 5 mg/mL, the absorption between 350 nm and 400 nm (essential absorption range of the blue phase) was distinct, although a considerable fraction of light was still transmitted and thus accessible for photoemission processes of the blue phase. However, at 50 mg/mL, light was virtually completely absorbed close to 400 nm. Accordingly, the emission of the blue phase was significantly reduced relative to that of the green phase at a concentration of 5 mg/mL and almost completely suppressed at 50 mg/mL, whereas the emission maximum of the green phase was hardly affected, even at the higher concentration.

## 4. Conclusions

Signal shifts in ^13^C NMR spectra of the aromatic region of PPM reveal the formation of complexes of silver(I) ions and tricarbonylchromium(0) by coordination to phenylene groups of PPM. In the case of silver, the interaction of silver(I) with PPM is further indicated by changes in UV-vis spectra and dissolution of otherwise insoluble silver salts (AgCF_3_SO_3_, AgClO_4_) in chloroform. Further evidence of chromium(0) complexes stems from ^1^H NMR spectra (new signal at around 5 ppm) and IR spectra (two strong peaks at 1962 cm^−1^ and 1884 cm^−1^, which are typical for tricarbonylchromium(0) moieties coordinated to arenes). A fraction of 5–10% metal centers per phenylene unit was present in the investigated complexes. In chloroform, peak broadening in PPM-silver(I) indicates dynamic processes, which are common for silver(I) interacting with arenes via η^2^ coordination. In contrast, η^6^ coordination of phenylene groups takes place with chromium(0) centers, with the Cr(CO)_3_ moieties localized, in line with the sharp signals in ^13^C NMR spectra.

The silver(I) and chromium(0) complexes are stable in chloroform, but in THF, the Ag^+^ complexes decay into PPM and Ag^+^. Second-order kinetics were identified in the decay of a complex with perchlorate as the counterion.

PPM remains fluorescent upon coordination. However, the so-called blue and green photoluminescent phases of PPM are differently affected by the coordinated metal centers. In the case of silver, the green phase is discriminated relative to the blue phase. For instance, the intensity of the absorption maximum of the green phase in PL spectra is about a factor of two lower relative to that of the blue phase compared to PPM alone. In the case of the chromium complexes, the very high absorption of the tricarbonylchromium(0) unit close to 400 nm strongly interferes with the photoemission of PPM at higher energies, particularly at higher concentrations. This leads to a suppression of the blue phase relative to the green phase. For example, in PLE spectra at concentrations of 50 mg/mL, the emission of the blue phase almost disappears, whereas the emission maximum of the green phase is hardly affected. Therefore, it is possible to change the perceived optical emission of PPM by the formation of metal complexes.

## Data Availability

The figures contain the relevant data referred to in this publication.

## Supplementary Materials

The following supporting information can be downloaded at: https://www.mdpi.com/article/10.3390/polym14173465/s1, Figure S1: IR spectra of (a) poly(phenylene methylene) (PPM), (b) PPM-Cr(CO)_3_, (c) PPM-AgCF_3_SO_3_, and (d) PPM-AgClO_4_; Figure S4: ^13^C NMR spectra of PPM (black), PPM-AgCF_3_SO_3_ (red) and PPM-AgClO_4_ (blue) in chloroform (a) and extended aromatic region (b), and in tetrahydrofuran (THF) (c) and extended aromatic region (d); Figure S5: ^1^H NMR spectra in chloroform, (a) PPM, (b) PPM-AgCF_3_SO_3_ and (c) PPM-AgClO_4_.
