# Supplementary material for: Studies on the Interaction of Poly(phenylene methylene) with Silver(I) and Hexacarbonylchromium(0)
Source: Polymers (Basel). 2022 Aug 25;14(17):3465. doi: 10.3390/polym14173465 (PMC9460465; doi:10.3390/polym14173465)
Supplement: Supplementary file 1 [file polymers-14-03465-s001.zip › polymers-1863421-SI.pdf]

Supplementary Material

Studies on the Interaction of Poly(Phenylene Methylene) with Silver(I) and Hexacarbonylchromium(0)

1. Infrared Spectra of PPM, PPM-Cr(CO)$_3$, PPM-AgCF$_3$SO$_3$ and PPM-AgClO$_4$

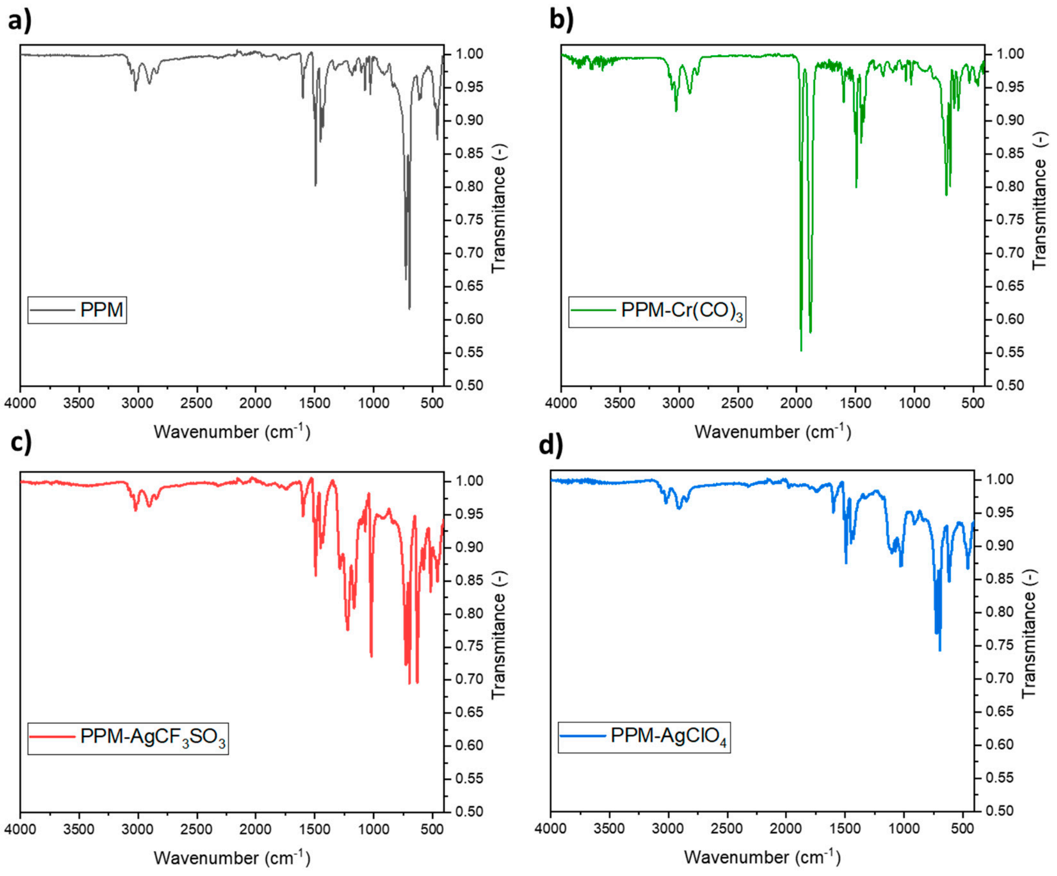

Figure S1. IR spectra of (a) poly(phenylene methylene) (PPM), (b) PPM-Cr(CO)$_3$, (c) PPM-AgCF$_3$SO$_3$, and (d) PPM-AgClO$_4$.

2. Quantitative Determination of Trifluoromethanesulfonate in PPM-$AgCF_3SO_3$ by IR Spectroscopy

For quantitative comparisons, first a linear baseline correction was performed on the basis of the two transmittance values at 4000 cm^{-1} and 3200 cm^{-1}, using the Formula (S1).

$$T_b = 1 + T - T_1 - (\nu - \nu_1)\frac{T_2 - T_1}{\nu_2 - \nu_1} \tag{S1}$$

with T_b the transmittance after baseline correction at the frequency ν, T the transmittance before baseline correction at the frequency ν, and T_1 and T_2 the transmittances at the frequencies ν_1 (4000 cm^{-1}) and ν_2 (3200 cm^{-1}), respectively. Subsequently, the peak at 1493 cm^{-1} in the spectrum of PPM itself was used as a reference for internal calibration. Importantly, the position of this peak was not affected in the products of PPM with the silver salts. Its transmittance was arbitrarily set to 0.8 and the transmittances at the other frequencies were adjusted accordingly, using the Formula (S2).

$$T_c = 1 - 0.2\frac{1 - T_b}{1 - T_{b,ref}} \tag{S2}$$

with T_c the transmittance after calibration at the frequency ν, and $T_{b,ref}$ the transmittance at the reference frequency (1493 cm^{-1}). The effects of baseline correction and calibration on the IR spectrum of PPM are illustrated on the example of PPM itself in Figure S2.

Calibrated IR spectra of PPM-$AgCF_3SO_3$ with different concentrations of the silver salt (1% w/w – 30% w/w and neat $AgCF_3SO_3$) are displayed in Figure S3. Figure S3 (c) shows that the intensity of five characteristic bands of the trifluoromethanesulfonate (1290 cm^{-1}, 1236 cm^{-1}, 1022 cm^{-1}, 633 cm^{-1} and 517 cm^{-1}) is proportional to the concentration of silver salt, thus allowing quantitative determination of the silver salt in PPM-$AgCF_3SO_3$ compositions.

It is also evident from Figure S3 that the bands of trifluoromethanesulfonate are sharper in the PPM-silver(I) materials than in silver(I) trifluoromethanesulfonate and also shifted to some extent, which may indicate that the interaction of trifluoromethanesulfonate with silver(I) is influenced by the coordination of the silver(I) ions in PPM.

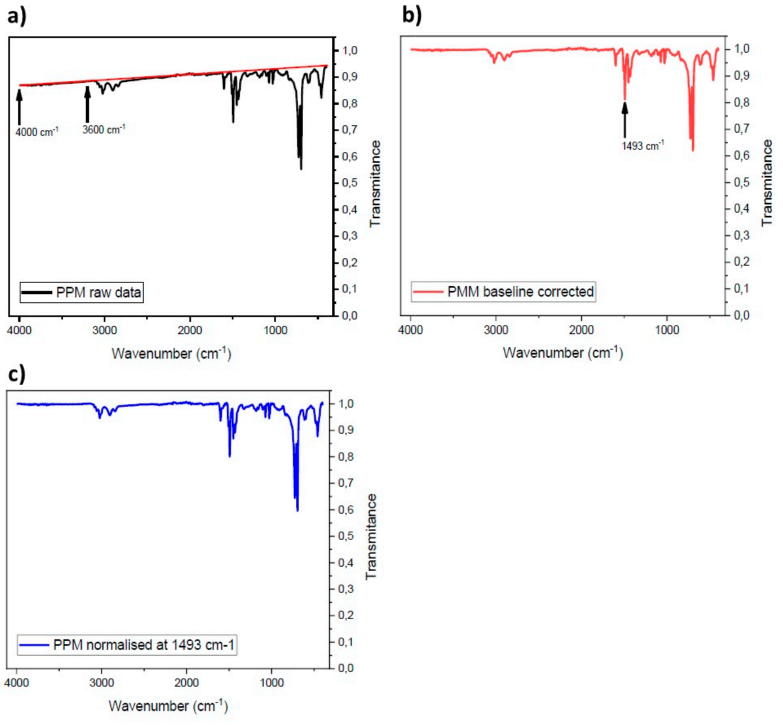

Figure S2. Successive data treatment of IR spectra of PPM. (a) spectrum as received, (b) spectrum with subtracted baseline, (c) normalized spectrum.

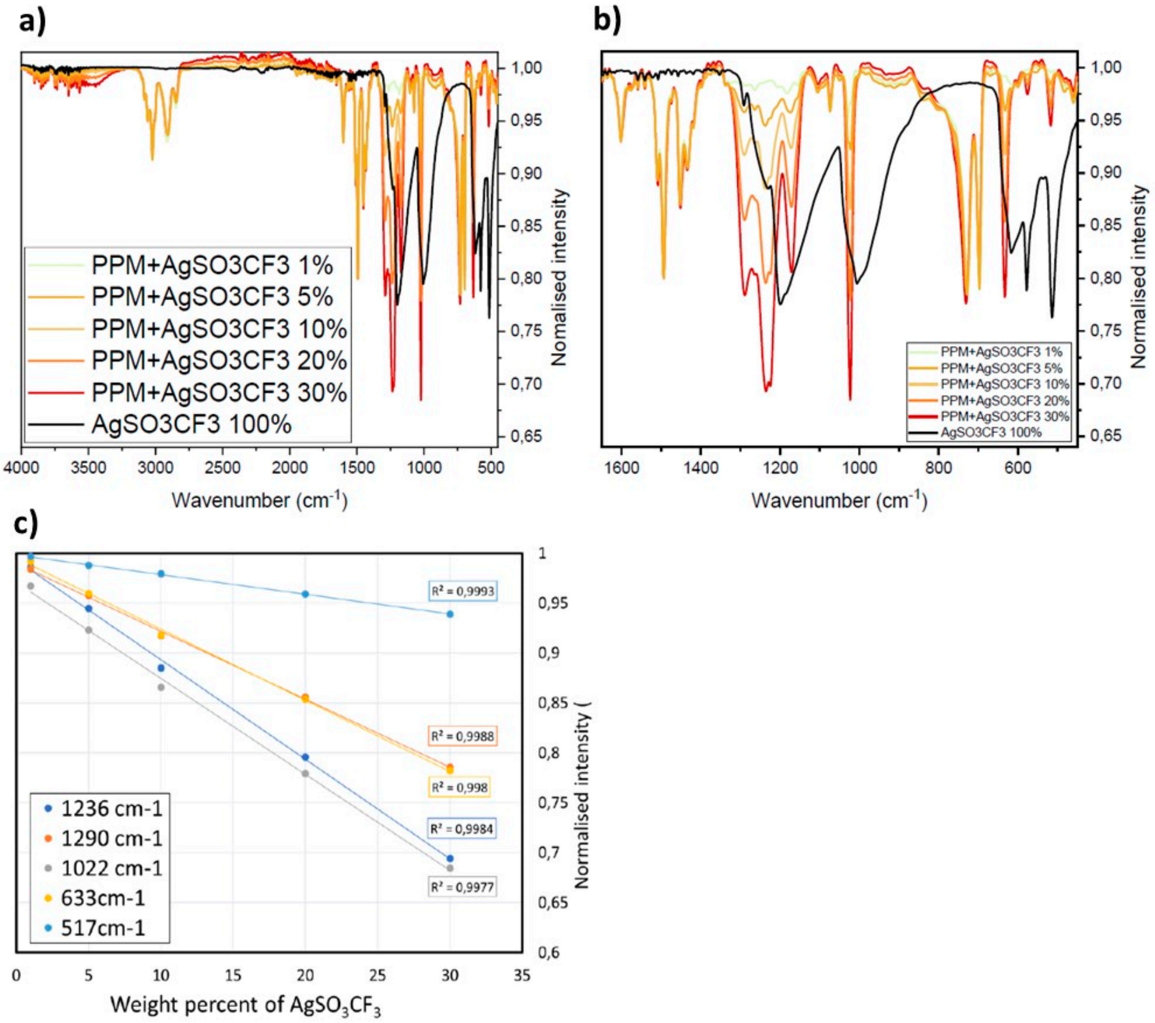

Figure S3. Influence of $AgSO_3CF_3$ salt concentration on IR spectra of PPM-$AgSO_3CF_3$. (a) Spectra normalized at 1493 cm^{-1} for weight percent in salt of 1 %, 5 %, 10 %, 20 %, 30 % and 100 % (i.e. the neat silver salt) for two wavenumber intervals (4000 cm^{-1} - 400 cm^{-1} and (b) 1650 cm^{-1} - 400 cm^{-1}). (c) Linear regression with regression coefficient R^2 for different characteristic peaks of trifluoromethanesulfonate: 1290 cm^{-1} (orange line and dots, R^2=0.999), 1236 cm^{-1} (dark blue line and dots, R^2=0.998), 1022 cm^{-1} (grey line and dots, R^2=0.998), 633 cm^{-1} (yellow line and dots, R^2=0.998), 517 cm^{-1} (blue line and dots, R^2=0.999).

3. ^{13}C-NMR spectra of PPM, PPM-$AgCF_3SO_3$ and PPM-$AgClO_4$

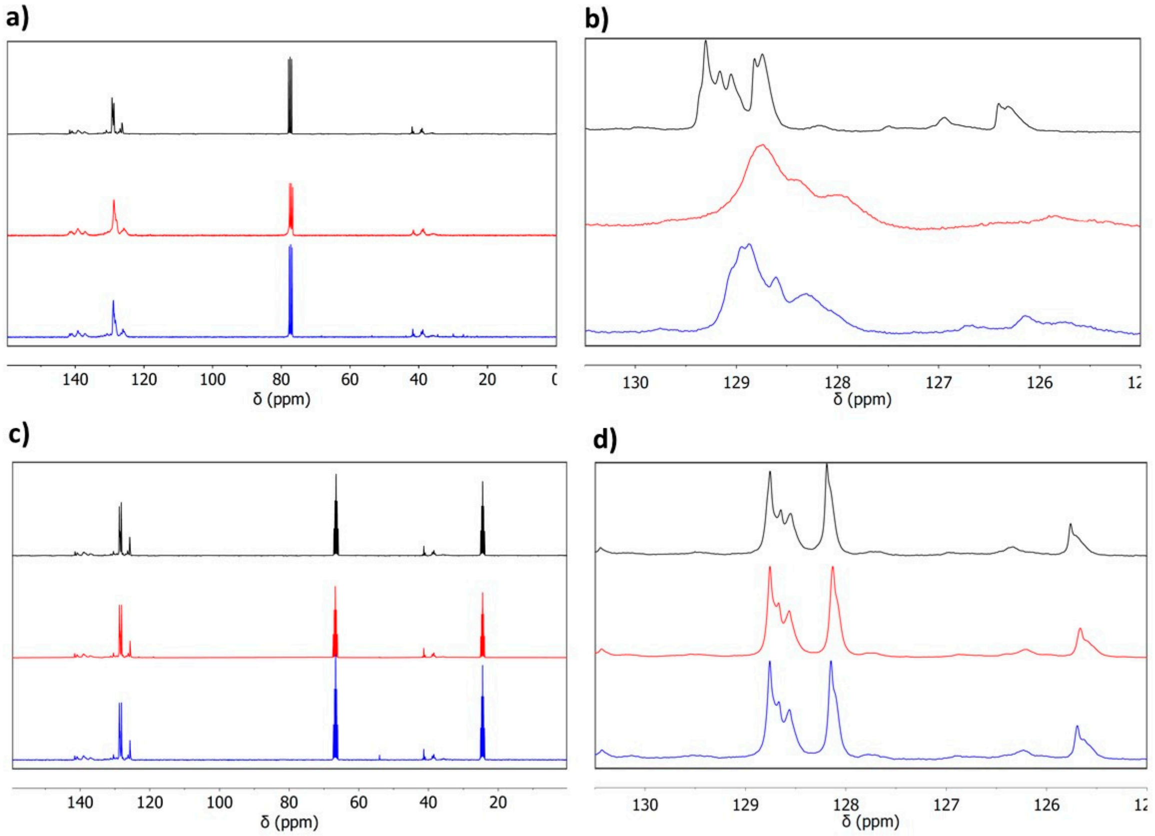

Figure S4. ^{13}C NMR spectra of PPM (black), PPM-$AgCF_3SO_3$ (red) and PPM-$AgClO_4$ (blue) in chloroform (a) and extended aromatic region (b), and in tetrahydrofuran (THF) (c) and extended aromatic region (d).

4. ^{1}H-NMR spectra of PPM, PPM-$AgCF_3SO_3$ and PPM-$AgClO_4$

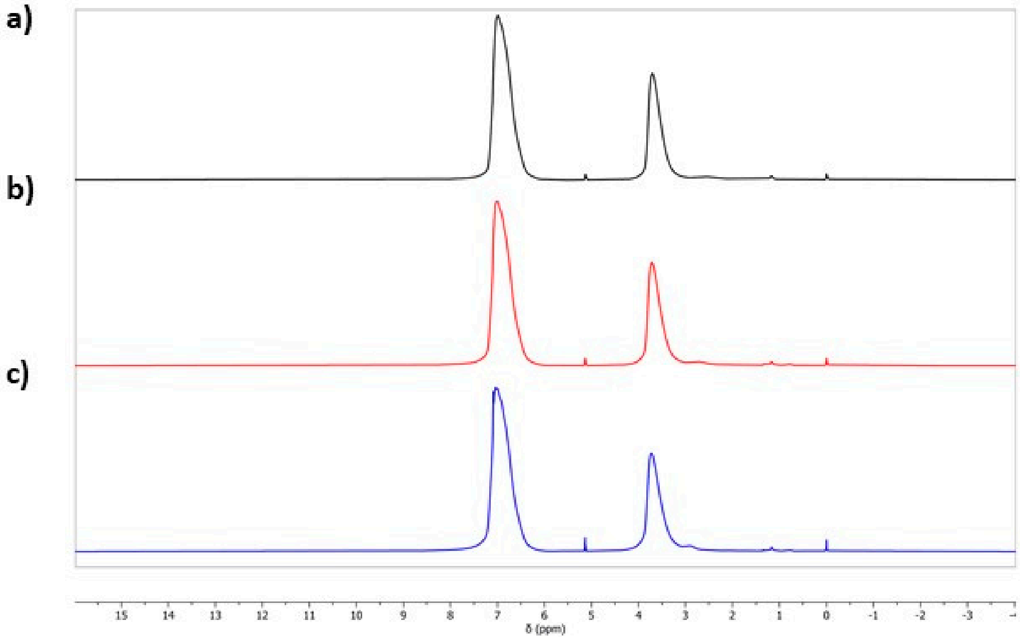

Figure S5. ^{1}H NMR spectra in chloroform, (a) PPM, (b) PPM-$AgCF_3SO_3$ and (c) PPM-$AgClO_4$.
